# The surgical management of the refractory overactive bladder

**DOI:** 10.4103/0970-1591.65402

**Published:** 2010

**Authors:** Nikhil Vasdev, Benjamin D. Biles, Raveen Sandher, Tahseen S. Hasan

**Affiliations:** Department of Urology, Freeman Hospital, Newcastle Upon Tyne, NE77DN, UK

**Keywords:** Refractory overactive bladder, surgical management

## Abstract

The refractory overactive bladder is a clinically challenging entity to manage and affects millions of people worldwide. Current surgical treatment options include botulinum toxin type A, sacral neuromodulation, and bladder reconstruction surgery all of which require careful attention to the individual patients needs and circumstances. In our paper we present a detailed up-to-date review on all the above mentioned surgical techniques from current literature and briefly describe our units experience with sacral neuromodulation.

## INTRODUCTION

Overactive bladder syndrome (OABS) is defined by the International Continence Society (ICS) as a symptom complex of lower urinary tract dysfunction.[1] These Overactive bladder (OAB) related lower urinary tract symptoms are prevalent in the community and continue to be a common cause of urological referrals worldwide.[2] The OAB symptom complex is defined by the ICS as urgency with or without urge urinary incontinence but usually with urinary frequency and nocturia.[3]

In a telephonic survey performed in the United States of America (USA), the overall prevalence of OAB wet was 9.6% in women over the age of 18 years rising from 5% in those aged 18-44 to 18% over the age of 65.[4] In the United Kingdom (UK), the Leicestershire Medical Research Council (MRC) incontinence study found the overall prevalence of OAB in women aged 40 and over to be 21.4%.[5] It is estimated that although patients with OAB might not seek a urological consult for symptoms, 20.4% of the population above the age of 40 years and over have a healthcare requirement.[6]

The OABS often causes poor bladder control resulting in complications such as increasing risk of falls/fractures in the elderly, depression, skin infections, and vulvovaginitis.[7,8] All these concomitant issues related to OABS such as urinary tract infections (UTI) increase health costs for patients receiving treatment.[8] The estimated health-related cost of managing OABS in the USA is approximately around $9bn (£5bn) per annum.[9] The emerging cost patterns clearly raise the possibility that early and definitive management of patients presenting with the OABS might both improve patient care and minimize the overall use of healthcare resources.[9]

The underlying etiology of OAB comprises both neurogenic and non-neurogenic detrusor dysfunction hence covering a somewhat broad spectrum of etiologies to treat.[10] We present a systematic review of current surgical management options for patients diagnosed with a refractory OAB in the UK and briefly present our experience with the permanent neuroprosthesis insertion for sacral neuromodulation (SNM).

## THE REFRACTORY OVERACTIVE BLADDER

Pharmacotherapy (anticholinergics) in conjunction with behavioral and dietary advice remains the mainstay of the initial management for most patients presenting with OABS. Anticholinergic medications, however, can cause mild to moderate side effects including dry mouth, gastro-intestinal disturbances including constipation, blurred vision, dry eyes, drowsiness, and skin reactions. Rarely, central nervous system stimulation can lead to restlessness, disorientation, hallucinations, and convulsions. Unwanted side effects of anticholinergenics and poor long-term compliance can be progressive issues in patients with symptomatic OAB.[11] The International Consultation on Incontinence (ICI) states that when the first-line approach is not (fully) satisfactory or fails after 8-12 weeks, alternative therapies including surgical management options should be considered.[3]

The main principles of further surgical intervention in patients with the refractory OAB is the utilization of procedures with a potential to achieve a reduction in bladder pressure, induce stabilization of overactivity, and increase bladder capacity. Current interventions include the use of intradetrusor botulinum toxin-A (BTX-A) injections and SNM. In more severe cases in which both the above-mentioned interventions fail to improve symptoms, the patient can be counseled on bladder reconstructive surgery such as urinary diversion, augmentation cystoplasty, or detrusor myomectomy.

## BLADDER BOTULINUM TOXIN A

Botulinum toxin (BTX) is a potent natural neurotoxin. It was first isolated in 1897 by Van Ermengem[12] who described the side effects of flaccid paralysis. BTX inhibits the release of acetylcholine at the presynaptic cholinergic junction, hence inducing muscle relaxation.[13] The toxin is derived from a gram-positive coccus *Clostridium Botulinum*. Several distinct structural serotypes of BTX have been identified (A, B, C, D, E, F, and G).[14] As urologist we commonly use the type ‘A’ stain of BTX for the management of patients with OAB.

When injected within the detrusor muscle, BTX is transmitted via endocytosis and is then bound via a synaptic vesical membrane proteins to trigger the exocytic process on membrane depolarization and calcium influx.[15] Three proteins located in the synaptic vesical membrane are crucial for BTX to function. These include synaptobrevin, vesicle-associated membrane protein (VAMP) and synaptosome-associated protein 25 kd (SNAP-25). These proteins are located on the plasma membrane of the presynaptic nerve terminal. Together these make up the soluble N-ethylmaleimide-sensitive factor attachment protein receptor (SNARE) complex. Disrupting the formation of this complex prevent exocytosis of the neurotransmitter, and stimulation of postsynaptic receptors (such as those on muscle) is prevented. BTX types A cleaves the SNAP-25 protein on the plasma membrane. When this occurs, the SNARE complex cannot form, vesicles containing Ach and other transmitters cannot dock to and fuse with the neuronal membrane, and neurotransmitters are not released. In the urinary bladder, BTX-A at the presynaptic cholinergenic junction induces detrusor muscle relaxation by inhibiting the release of acetylcholine from the presynaptic nerve terminal.[16] Hence, when patients receive intradetrusor BTX-A injections, affected muscarinic receptors in the detrusor muscle cannot be stimulated and detrusor voluntary contractions are reduced/suppressed.[15]

In 1999, Stroher and colleagues[17] first described the use of BTX-A in the treatment of neurogenic OAB. Since 1999, there has been a gradual steady increase in the use of BTX-A in the managements of patients with refractory OAB.[18] Bladder injections of BTX-A can be performed using either a flexible or rigid cystoscope under local, spinal or general anesthesia. The dose injected ranges from 100 to 300 units in one sitting and is generally injected into 10-40 sites within the detrusor wall[19]. Presently, the clinical usage of intravesical BTX-A injections await further evaluation with regards to optimum dosage, site, and number of injections.

In the current literature, there are only a few series evaluating the role of BTX-A in the refractory OAB. These studies, however, report observing a significant improvement in urinary frequency, initial improvement in leakage episodes, and cure/improvement rates of 60-75% at a varying follow up of 3-6 months, in the patient population studied.[20–22] Significant improvements were noted with symptoms of OAB, urodynamic variables, and quality of life at 12 weeks after intradetrusor injections of 200 U of BTX-A in 16 patients in comparison to 18 patients in the placebo arm.[23] Current trials, however, are based on relatively small numbers of patients[24] and larger regulatory trials are awaited in the future. The adverse effects and complications reported in the current literature are seen in approximately 10% of patients who have had intradetrusor BTX-A for the refractory OAB. Some of the commonly reported complications include hematuria, pelvic pain, transient dysuria, transient retention, difficulty in urination, and UTIs. The literature review also suggests that BTX-A can cause significant increases in the post-micturation residual volumes resulting in the need for clean intermittent self-catheterization (CISC) in some patients. The willingness of patients to perform CISC, if necessary, should therefore be assessed prior to proceeding with intravesical botulinum toxin therapy. We summarize the details of recent publications outlining the use of BTX-A in patients with refractory OAB[20–22,25–28] in Table 1.

**Table 1:** The use of intradetrusor BTX-A therapy in patients with refractory OAB

Author	Number of patients	BTX-A dose	Number of injection sites in bladder	Efficacy (response / response Rate) (%/N)	Adverse effects (%/N)	CISC post BTX-A (%/N)
Rapp[20]	35	300 U	30	60 (35) - improvement of voiding symptoms after 3 weeks lasting upto 6 months	Hematuria, pelvic pain, and dysuria	None reported
Kuo[21]	20	200 U	20	45 (9)- improvement in continence at 3 months	Haematuria, UTI in	30 (6)
				15 (3) – failed to respond	35 (7)	
Werner[22]	26	100 U	30	76 (20) –	UTI in 34 (9)	7 (2)
				Improvement after 4-12 weeks of treatment		
Schulte-Baukloh[25]	44	200 –	20 – 30	86 (38) –	None reported	None reported
		300 U		Improvements in symptoms and urodynamic findings		
Schulte-Baukloh[26]	7	300 U	30	71 (5) – improvement in symptoms and bladder capacity at 3 months. The overall satisfaction score (on a scale of 0 to 10) averaged 6.8.	None reported	None reported
Flynn[27]	10	150 U	20	50 (5) – improvement in symptoms at 3 months.	UTI in 33 (3)	None reported
Rajkumar[28]	15	300 U	30	46 (14) – improvements in symptoms at 3 months.	None reported	None reported

## SACRAL NEUROMODULATION

In 1940 and 1941, Dees[29] studied bladder contraction followed by activation of the pelvic nerves. In the late 1960s, Nashold[30] commenced experiments with spinal cord stimulation. This group compared the effectiveness of stimulating the dorsal surface of the spinal marrow in acute and chronic studies. It was noted that surface stimulation at the level of S2 resulted in the generation of high intravesical pressures, although no voiding was noted. Bladder stimulation and emptying was only achieved when bipolar electrodes were placed in the central gray matter of S1-S2. In 1972, Nashfold[31] reported the use of sacral stimulation in four patients. In a subsequent review of 27 neuroprosthesis used over 10 years, the success rate was 55.6%.[32] Following initial assessments, the Food and Drug administration (FDA) authorized the use of the SNM device in 1997. SNM is now recommended to be a safe, effective, minimally invasive urological surgical technique for the treatment of a diverse spectrum of lower urinary tract diagnosis including the refractory OAB. Additional usages of SNM, in Urology, include disorders such as urinary urge incontinence, dysfunctional voiding, and idiopathic urinary retention.[33]

Normal micturation depends on intact neural pathways in the central and peripheral nervous systems. The function of the urinary bladder and urethral sphincters requires co-ordination in order to achieve a low pressure micturation, thus maintaining safety of the urinary tract. Voiding in infancy traditionally is reflexive with the brain having a passive role in the co-ordination of micturation. In adulthood, timing reflects volitional control with neurological development. This subsequently leads onto an ability to initiate voiding over a wide range of bladder volumes. In normal adults, the bladder appears to exhibit a predominantly voluntary rather than involuntary (autonomic) neural control. An additional important function is the ‘All or none’ manner to initiate voiding and hence emptying the bladder completely. The positive feedback to the bladder to initiate micturation is mediated by the parasympathetic pathways to the higher micturation centers. In patients with the refractory OAB, it is possible that this feedback system is disturbed leading onto the development of detrusor overactivity and related symptoms. Within the spinal cord, the primitive sacral centre can help regulate this feedback activity, hence the centre of application of SNM. SNM therapy relies on electrical stimulation to modulate signal transmission predominantly involving the somatic afferent axons in the spinal nerve roots. These in turn influence voiding and continence reflex pathways in the central nervous system. In patients with refractory OAB, it is possible, therefore, to inhibit detrusor hyperactivity by direct stimulation of the bladder preganglionic neurons and inhibition of interneuronal transmission in the afferent limb of the micturation reflex.

In clinical practice, all patients being evaluated for SNM would have previously received a number of conservative treatment options for their OABS including anticholinergenic medications, pelvic floor physiotherapy, if indicated, and behavioral modification therapy with inadequate symptomatic benefit. The SNM procedure involves a two-phase approach. In the first phase, selected patients undergo a short period (7-10 days) of neuromodulation with a view to assess the impact of electrical stimulation on lower urinary tract symptoms. This phase involves the placement of an electrode into the S2 or S3 sacral foramen coupled to an external pulse generator, under local or general anesthesia. Responding patients (>50% symptomatic benefit) then proceed to the second phase which involves the implantation of a permanent neuroprosthesis, usually requiring a general anesthesia. Careful preoperative counseling, meticulous patient selection, and expression of sensitivity in management of patent expectations remain critical toward a successful outcome of this procedure.

Recently, the National Institute for Clinical Excellence (NICE, UK) interventional procedures (IP) guidance aimed to evaluate the efficacy and safety of permanent SNS for the refractory OAB.[34] This document incorporated current systematic reviews[35,36] including case reports. Currently, three randomized control trials (RCT) are present in the literature of which two have been conducted by the sacral nerve stimulation group[37,38] evaluating patients. Analysis of these RCT’s reveals an overall failure rate of 21% over a median follow-up period of 18 months (range of 6-36 months). Common patients’ side effects were pain at the implant site, lead migration, and leg pain. Other side effects included leg stimulation, disturbed bowel habits, urinary retention, and skin irritation at the implant site. Of the total 157 patients enrolled across the sacral nerve stimulation group, 33% had adverse effects that required surgical revision. Current, recommendation in the UK NICE guidelines mention that upto two-thirds of patients achieve continence or substantial improvement in symptoms after SNS and the available data show that beneficial effects appear to persist for upto 3-5 years after implantation. About one-third of patients may require re-operation attributed to pain at the implantation site, infection or the need for adjustment and modification of the lead system. Permanent removal of the electrodes may be required in one in ten patients. Additionally, lifelong follow up in all these patients is required. Results of some recently published papers are summarized in Table 2.[35–42]

**Table 2:** The use of sacral neuromodulation for treatment of patients with refractory OAB

Author	Number of patients	Type of technique for SNM	Outcome	Complications (%)	Follow up (months)	Removal / failure rate (%)
Brazzelli[35]	120 (Review)	PNE	-80 achieved continence or greater than 50 improvement in their main incontinence symptoms	Pain at implant site- 25 Lead migration - 16, Replacement and repositioning of the implanted pulse generator in 15, Wound problems in 7, Adverse effects on bowel function in 6, Infection in 5 Generator problems in 5.	36-60	9
Latini[36]	41	Two staged implant	-90 of patients had 50 or greater improvement in presenting symptoms and quality-of-life parameters	Pain at implant site- 29	12-24	7
				Lead migration - 5,		
				Wound infection – 15		
Hassouna[37]	25	PNE	-56 of patients improved with at-least a 50 improvement in their main incontinence symptoms	None reported	6-24	None reported
Siegel[38]	29	PNE	-56 of patients improved with at-least a 50 improvement in their main incontinence symptoms	None reported	24	None reported
			-A 69 of patients improved			
Weil[39]	21	PNE	-88 of patients improved	Pain at implant side – 20	6-36	32
Schmidt[40]	58	PNE	- 47 of patient improved	Pain at implant site - pulse 15.9	6-36	33
			-30 of patients noticed a improvements in all symptoms by 50	Lead migration - 7.0		
Spinelli[41]	196	Two staged implant	-45-65 improvement in symptoms	None reported	6-18	None reported
Everaert[42]	53	Two staged implant	-85 improvement in symptoms	Pain at implant site - pulse 20	12-24	None reported

In our unit between February 2001 and July 2008, 28 patients (19 females and 9 males) with a mean age of 47 years (range 22-72) underwent a two stage permanent sacral neuroprosthesis implantation. All 28 patients were diagnosed with a refractory OAB prior to the procedure. The mean number of PNE’s used in each patient to evaluate clinical use of SN was 2.1 (1-3). At a mean follow up of 37.9 ± 5.1 months (range 0.8-95.3 months), six patients (21.4%) failed to notice a significant improvement in symptoms requiring removal of the permanent neuroprosthesis in four of the six patients. The remaining 22 patients (78%) continue to be satisfied with their permanent neuroprosthesis at their respective last follow-p appointment. We will in due course publish details on the above-mentioned data.

In conclusion, SNM is a unique and fully reversible treatment option for patients with refractory OAB. This technique can also be used effectively in patients with urinary incontinence, urinary retention, fecal incontinence, and pelvic floor dysfunction.[24] Overall, current data indicate that an estimated 70% of patients with refractory OAB who receive SNM show an improvement with symptoms.

## BLADDER RECONSTRUCTIVE SURGERY

The main principles of any reconstructive bladder surgery for refractory OAB involve enhancement of functional bladder capacity and reduction in spontaneous increments in intravesical pressure. Augmentation entero-cystoplasty and detrusor myectomy are two bladder reconstructive surgical procedures which are often performed for patients with refractory OABS with the potential of achieving the above aims.

In augmentation cystoplasty, the bladder capacity is increased by bivalving the bladder wall and replacing it with a segment of bowel. Incorporation of bowel segment also has the potential of diminishing detrusor contractility. In clinical practice, ileum is often the most commonly used segment of bowel in adult patients. No current randomized controlled trials, however, are available in the literature in order to evaluate the role of augmentation cystoplasty for the management of refractory OAB. A few case series in the current literature evaluate augmentation cystoplasty in patients with refractory OAB; however, within these papers most patients had additional pathologies such as interstitial cystitis[43] or stress urinary incontinence[44] rendering comparative analysis unreliable.

Our own experience in the use of augmentation enterocystoplasty in patients with refractory OAB has been encouraging.[44] We evaluated the role augmentation cystoplasty in 48 patients of whom 35 (73%) patients had a refractory OAB. Early symptomatic outcome was good in 40 (83%) patients, moderate in 7 (15%) and unsatisfactory in 1 (2%) patient. The mean symptom scores before and 3 months after surgery were 10 (range 2-14) and 3 (range 2-14), respectively (*P* < 0.001). There was a significant increase in total bladder capacity (307 ± 140 to 588 ± 217 mL; *P* < 0.001) and bladder compliance (37 ± 50 to 169 ± 162 mL/cm H_2_O; *P* < 0.001). Clean intermittent self-catheterization (CISC) was performed by 36 (75%) patients. On urodynamic analysis, detrusor overactivity persisted in 15 (31%) patients. Quality of life scores revealed significant improvements in all domains. Late complications (> 30 days) included incisional hernia (3), anastomotic perforation (1), calculus formation (1), and urethral stricture (1). The long-term outcome was good or moderate in 12 patients (92%) with neurogenic bladder dysfunction and good or moderate in 19 patients (58%) with DO.

On reviewing the additionally published literature on augmentation cystoplasty, the complication rate associated with the procedure continues to be high including the specific side effects of recurrent UTI’s, mucus retention, urinary tract calculus formation, metabolic disturbances, long-term deterioting renal function, and risk of bladder perforation.[45] In view of the potential side effects associated with the procedure, patients must be counseled in detail prior to surgery and must be assessed for suitability to commence CISC. A recent case report[46] describes the use of successful SNM in patients with OAB refractory to bladder augmentation.

Detrusor myectomy aims to improve bladder function by excising bladder muscle from the fundus of the bladder while leaving the bladder mucosa intact. The segment is commonly covered with omentum which carries the potential of creating a permanent wide-neck diverticulum. All current cases reports in the literature indicate an unclear stratification in clinical improvement; hence this procedure is not well established.[47]

For some patients with refractory OAB, creation of an ileal conduit urinary diversion remains another viable option, particularly for those who might be deemed unsuitable for reconstruative bladder surgery. The procedure, however, is not without complications including the risks of recurrent urinary sepsis, and upper tract dilatation, and the possibility of renal function deterioration in the longer term.[48] Patient information, counseling, and careful selection clearly remain mandatory for successful outcome.

## CONCLUSION

OABS affects millions of people worldwide with an increase in symptom prevalence with advancing population ages. Conventional therapy for OABS includes behavioral modification and use of anticholinergic medications. In some patients the symptom complex might pursue a protracted course with the potential of severely affecting the overall quality of life in these individuals.

Management of patients with refractory OAB can be clinically challenging and requires careful attention to individual patients needs and circumstances.

Over recent years, a number of minimally invasive procedures such as SNM and the use of intravesical botulinum toxin injection have emerged with encouraging results in patients with refractory OABS. Our own experience with these procedures has also been very positive and clearly indicates a careful use of these techniques in selected patients with refractory OABS prior to proceeding with irreversible major surgical interventions such as entercystoplasty or urinary diversion.

## References

[1] Wein AJ, Rackley RR (2006). Overactive bladder: A better understanding of pathophysiology, diagnosis and management. J Urol.

[2] Tyagi S, Thomas CA, Hayashi Y, Chancellor MB (2006). The overactive bladder: Epidemiology and morbidity. Urol Clin North Am.

[3] Abrams P, Artibani W, Cardozo L, Dmochowski R, van Kerrebroeck P, Sand P (2009). International Continence Society: Reviewing the ICS 2002 terminology report: The ongoing debate. Neurourol Urodyn.

[4] Stewart WF, Van Rooyen JB, Cundiff GW, Abrams P, Herzog AR, Corey R (2003). Prevalence and burden of overactive bladder in the United States. World J Urol.

[5] McGrother CW, Donaldson MM, Hayward T, Matthews R, Dallosso HM, Hyde C (2006). Leicestershire MRC Incontinence Study Team. Urinary storage symptoms and comorbidities: A prospective population cohort study in middle-aged and older women. Age Ageing.

[6] McGrother CW, Donaldson MM, Shaw C, Matthews RJ, Hayward TA, Dallosso HM (2004). Storage symptoms of the bladder: Prevalence, incidence and need for services in the UK. BJU Int.

[7] Teleman PM, Lidfeldt J, Nerbrand C, Samsioe G, Mattiasson A (2004). WHILA study group. O7men. BJOG.

[8] Darkow T, Fontes CL, Williamson TE (2005). Costs associated with the management of overactive bladder and related comorbidities. Pharmacotherapy.

[9] Hu TW, Wagner TH (2005). Health-related consequences of overactive bladder: An economic perspective. BJU Int.

[10] Hampel C, Gillitzer R, Pahernik S, Hohenfellner M, Thüroff JW (2003). Epidemiology and etiology of overactive bladder. Urologe A.

[11] Chapple CR, Khullar V, Gabriel Z, Muston D, Bitoun CE, Weinstein D (2008). The effects of antimuscarinic treatments in overactive bladder: An update of a systematic review and meta-analysis. Eur Urol.

[12] Erbguth FJ (2004). Historical notes on botulism, Clostridium botulinum, botulinum toxin, and the idea of the therapeutic use of the toxin. Mov Disord.

[13] Simpson LL (1986). Molecular pharmacology of botulinum toxin and tetanus toxin. Annu Rev Pharmacol Toxicol.

[14] Dmochowski R, Sand PK (2007). Botulinum toxin A in the overactive bladder: Current status and future directions. BJU Int.

[15] Nitti VW (2006). Botulinum toxin for the treatment of idiopathic and neurogenic overactive bladder: State of the art. Rev Urol.

[16] Apostolidis A, Popat R, Yiangou Y, Cockayne D, Ford AP, Davis JB (2005). Decreased sensory receptors P2X3 and TRPV1 in suburothelial nerve fibers following intradetrusor injections of botulinum toxin for human detrusor overactivity. J Urol.

[17] Stohrer M, Schurch B, Kramer G (2000). Botulinum A-toxin in the treatment of detrusor hyperreflexia in spinal cord patients: A new alternative to medical and surgical managements procedures?. Neurol Urodyn.

[18] Kim DK, Thomas CA, Smith C, Chancellor MB (2006). The case for bladder botulinum toxin application. Urol Clin North Am.

[19] Smith CP, Nishiguchi J, O’Leary M, Yoshimura N, Chancellor MB (2005). Singleinstitution experience in 110 patients with botulinum toxin A injection into bladder or urethra. Urology.

[20] Rapp DE, Lucioni A, Katz EE, O’Connor RC, Gerber GS, Bales GT (2004). Use of botulinum-A toxin for the treatment of refractory overactive bladder symptoms: An initial experience. Urology.

[21] Kuo HC (2004). Urodynamic evidence of effectiveness of botulinum A toxin injection in treatment of detrusor overactivity refractory to anticholinergic agents. Urology.

[22] Werner M, Schmid DM, Schüssler B (2005). Efficacy of botulinum-A toxin in the treatment of detrusor overactivity incontinence: A prospective nonrandomized study. Am J Obstet Gynecol.

[23] Sahai A, Dasgupta P, Khan MS (2007). Is botulinum A toxin an effective treatment for idiopathic detrusor overactivity and sensory urgency?. Nat Clin Pract Urol.

[24] Chapple C, De Ridder D (2009). The second-line management of idiopathic overactive bladder: What is the place of sacral neuromodulation and botulinum toxin-A in contemporary practice?. BJU Int.

[25] Schulte-Baukloh H, Weiss C, Stolze T, Herholz J, Stürzebecher B, Miller K (2005). Botulinum-A toxin detrusor and sphincter injection in treatment of overactive bladder syndrome: Objective outcome and patient satisfaction. Eur Urol.

[26] Schulte-Baukloh H, Weiss C, Stolze T, Stürzebecher B, Knispel HH (2005). Botulinum-A toxin for treatment of overactive bladder without detrusor overactivity: Urodynamic outcome and patient satisfaction. Urology.

[27] Flynn MK, Webster GD, Amundsen CL (2004). The effect of botulinum: A toxin on patients with severe urge urinary incontinence. J Urol.

[28] Rajkumar GN, Small DR, Mustafa AW, Conn G (2005). A prospective study to evaluate the safety, tolerability, efficacy and durability of response of intravesical injection of botulinum toxin type A into detrusor muscle in patients with refractory idiopathic detrusor overactivity. BJU Int.

[29] Dees JE (1965). Contraction of the urinary bladder produced by electric stimulation a preliminary report. Invest Urol.

[30] Nashold BS, Friedman H, Boyarsky S (1971). Electrical activation of micturition by spinal cord stimulation. J Surg Res.

[31] Nashold BS, Friedman H, Glenn JF, Grimes JH, Barry WF, Avery R (1972). Electromicturition in paraplegia. Implantation of a spinal neuroprosthesis. Arch Surg.

[32] Nashold BS, Friedman H, Grimes J (1982). Electrical stimulation of the conus medullaris to control bladder emptying in paraplegia: A ten-year review. Appl Neurophysiol.

[33] Leng WW, Chancellor MB (2005). How sacral nerve stimulation neuromodulation works. Urol Clin North Am.

[34] (2004). IPG64 Sacral nerve stimulation for urge incontinence: Understanding NICE guidance.

[35] Brazzelli M, Murray A, Fraser C (2006). Efficacy and safety of sacral nerve stimulation for urinary urge incontinence: A systematic review. J Urol.

[36] Latini JM, Alipour M, Kreder KJ (2006). ficacy of sacral neuromodulation for symptomatic treatment of refractory urinary urge incontinence. Urology.

[37] Hassouna MM, Siegel SW, Nÿeholt AA, Elhilali MM, van Kerrebroeck PE, Das AK (2000). Sacral neuromodulation in the treatment of urgency-frequency symptoms: A multicenter study on efficacy and safety. J Urol.

[38] Siegel SW, Catanzaro F, Dijkema HE, Elhilali MM, Fowler CJ, Gajewski JB (2000). Long-term results of a multicenter study on sacral nerve stimulation for treatment of urinary urge incontinence, urgency-frequency, and retention. Urology.

[39] Weil EH, Ruiz-Cerdá JL, Eerdmans PH, Janknegt RA, Bemelmans BL, van Kerrebroeck PE (2000). Sacral root neuromodulation in the treatment of refractory urinary urge incontinence: A prospective randomized clinical trial. Eur Urol.

[40] Schmidt RA, Jonas U, Oleson KA, Janknegt RA, Hassouna MM, Siegel SW (1999). Sacral nerve stimulation for treatment of refractory urinary urge incontinence: Sacral Nerve Stimulation Study Group. J Urol.

[41] Spinelli M, Bertapelle P, Cappellano F, Zanollo A, Carone R, Catanzaro F (2001). Chronic sacral neuromodulation in patients with lower urinary tract symptoms: Results from a national register. J Urol.

[42] Everaert K, De Ridder D, Baert L, Oosterlinck W, Wyndaele JJ (2000). Patient satisfaction and complications following sacral nerve stimulation for urinary retention, urge incontinence and perineal pain: A multicenter evaluation. Int Urogynecol J Pelvic Floor Dysfunct.

[43] Awad SA, Al-Zahrani HM, Gajewski JB, Bourque-Kehoe AA (1998). Long-term results and complications of augmentation ileocystoplasty for idiopathic urge incontinence in women. Br J Urol.

[44] Hasan ST, Marshall C, Robson WA, Neal DE (1995). Clinical outcome and quality of life following enterocystoplasty for idiopathic detrusor instability and neurogenic bladder dysfunction. Br J Urol.

[45] Greenwell TJ, Venn SN, Mundy AR (2001). Augmentation cystoplasty. BJU Int.

[46] Rasmussen NT, Guralnick ML, O’Connor RC (2009). Successful use of sacral neuromodulation after failed bladder augmentation. Can Urol Assoc J.

[47] Kumar SP, Abrams PH (2005). Detrusor myectomy: Long-term results with a minimum follow-up of 2 years. BJU Int.

[48] Singh G, Wilkinson JM, Thomas DG (1997). Supravesical diversion for incontinence: A long-term follow-up. Br J Urol.

